# Trajectory Definition with High Relative Accuracy (HRA) by Parametric Representation of Curves in Nano-Positioning Systems

**DOI:** 10.3390/mi10090597

**Published:** 2019-09-10

**Authors:** Lucía Díaz Pérez, Beatriz Rubio Serrano, José A. Albajez García, José A. Yagüe Fabra, Esmeralda Mainar Maza, Marta Torralba Gracia

**Affiliations:** 1 Aragon Institute of Engineering Research (I3A), University of Zaragoza, C/María de Luna 3, 50018 Zaragoza, Spain; 2 Department of Applied Mathematics, University Research Institute of Mathematics and its Applications(IUMA), University of Zaragoza, C/María de Luna, 3, 50018 Zaragoza, Spain; 3 Centro Universitario de la Defensa, Ctra. Huesca s/n, 50090 Zaragoza, Spain

**Keywords:** nano-positioning, CAD/CAM systems, high relative accuracy (HRA)

## Abstract

Nanotechnology applications demand high accuracy positioning systems. Therefore, in order to achieve sub-micrometer accuracy, positioning uncertainty contributions must be minimized by implementing precision positioning control strategies. The positioning control system accuracy must be analyzed and optimized, especially when the system is required to follow a predefined trajectory. In this line of research, this work studies the contribution of the trajectory definition errors to the final positioning uncertainty of a large-range 2D nanopositioning stage. The curve trajectory is defined by curve fitting using two methods: traditional CAD/CAM systems and novel algorithms for accurate curve fitting. This novel method has an interest in computer-aided geometric design and approximation theory, and allows high relative accuracy (HRA) in the computation of the representations of parametric curves while minimizing the numerical errors. It is verified that the HRA method offers better positioning accuracy than commonly used CAD/CAM methods when defining a trajectory by curve fitting: When fitting a curve by interpolation with the HRA method, fewer data points are required to achieve the precision requirements. Similarly, when fitting a curve by a least-squares approximation, for the same set of given data points, the HRA method is capable of obtaining an accurate approximation curve with fewer control points.

## 1. Introduction

Current trends in precision engineering demand high accuracy positioning systems that can be used for measuring and manufacturing applications [1]. For these, the meteorological challenge is an obstacle to the achievement of high accuracy, repeatability, and stability at sub-micrometer and nanometre scales over long travel ranges [2]. In these systems, the positioning error is minimized by implementing precision engineering design principles. In addition, the control system performance must be optimized to reduce the positioning uncertainty. In this line of research, at the University of Zaragoza, a novel nanopositioning platform (NanoPla) has been designed, built, and implemented [3]. The NanoPla requirements imply sub-micrometer accuracy in a large working range of 50 mm × 50 mm.

In some nanomanufacturing applications, nanopositioning stages are required to follow a predefined trajectory with a sub-micrometer precision [4]. The definition of a trajectory in traditional manufacturing systems, such as machine tools, proceeds as follows: First, the geometry is defined in a CAD program; then, this geometry is exported to a CAM program, where the machine and the toolpath generation conditions are set. However, simpler geometries can be directly designed in the CAM program. In addition, some programs include CAM and CAD functions within the same software. Finally, by means of a post-processor, a specific numerical control (NC) program is created [5]. Nevertheless, creating a complex curve in a CAD/CAM system is not always straightforward [6,7,8]. In CAD/CAM systems, complex curve trajectories are usually defined by curve fitting, having as input a set of points belonging to the nominal curve trajectory. The resultant fitting curve produces an equation that can compute points anywhere along the trajectory. This equation is determined by a set of control points together with the fitting method basis. However, trajectory definition by curve fitting in CAD/CAM systems results in fitting errors that, even though in traditional manufacturing systems are negligible, in nanopositioning stages can contribute greatly to the final positioning uncertainty of the stage.

In CAD/CAM systems, curve fitting is commonly performed by interpolation or by least-squares approximation. There are many types of interpolation methods, such as linear, circular, and polynomial interpolation [9]. Linear interpolation results in polylines, inducing large geometrical deviations that can only be reduced at the expense of the amount of data. On the contrary, spline interpolation can transform a large number of linear blocks into just a few spline blocks. In spline interpolation, the resultant curve depends on the control points obtained. When the trajectory is obtained by spline interpolation of a great number of interpolation points, a great number of control points is needed and the computational complexity increases. On the other hand, the curve obtained by a spline least-squares approximation does not necessarily contain all the given data points, but only approximately fits them. The number of control points that define the approximation curve is related to how close the resultant curve fits the given data points. Nevertheless, control points may be redundant or inadequate. Redundant control points unnecessarily increase the computational complexity without decreasing the fitting errors, whereas inadequate control points increase the fitting errors and, thus, the curve fails to satisfy the precision requirements [10]. Thus, the challenge of curve fitting, either by interpolation or approximation, lies in obtaining the most accurate trajectory, without compromising the computational efficiency.

Other works [11] have focused on optimizing trajectory generation for a smooth interpolating motion, in order to respect the machine kinematic feed-rate and acceleration limits, as well as to avoid fluctuations due to discontinuities in the first derivatives along the tool path. Spline-type interpolation has been applied in high-speed machining to limit the speed discontinuities while respecting the contour tolerance [10,12]. The target of these works is to achieve a confined error and minimal machining time; however, this task becomes harder for more accurate tolerances.

In the NanoPla application, speed and acceleration control is not as critical as accurately following a predefined trajectory. Therefore, an accurate curve fitting method capable of offering high accuracy in position definition along the whole trajectory is required. This work presents a novel method for accurate curve fitting with shape-preserving representations, allowing high relative accuracy (HRA) in the computations. Performing an algorithm with HRA is a very desirable goal, because it implies that the relative errors of the computations will be of the order of the machine precision, independent of the size of the problem. The proposed method, which will be called the HRA method, is based on recent advances in numerical linear algebra (see [13] and the references therein). This method considerably improves the precision in curve fitting and, thus, minimizes the contribution of the trajectory fitting errors in the total positioning uncertainty of a nanopositioning stage, such as the NanoPla. The obtained fitting trajectories have continuous successive derivatives, which can also be efficiently evaluated. In this work, curve fitting is performed by interpolation and by least-squares approximation. The HRA method has been developed taking into account both cases. In this article, the contribution to the final positioning error of the NanoPla in terms of the fitting errors of the novel HRA method is compared to the contribution of the fitting errors obtained by traditional CAD/CAM systems, considering also the number of data points and control points required to define the resultant curve, in each case. In addition, the relevance of the resultant fitting errors of each method, in terms of the final positioning error of the NanoPla, is experimentally analyzed.

## 2. Curve Fitting in Computer Aided Geometric Design (CAGD)

Due to the importance of curve definition in this work, this section briefly explains the procedures for defining a curve by interpolation or by least squares approximation.

**Figure 1:**
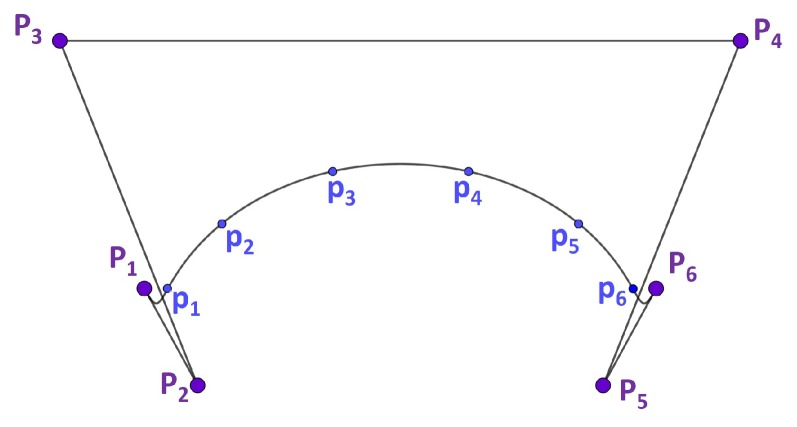
Curve fitting by interpolation, where $p_{1},p_{2},p_{3},p_{4},p_{5},p_{6}$ are the given data and $P_{1},P_{2},P_{3},P_{4},P_{5},P_{6}$ are the control points.

Interpolation is a fundamental tool in CAGD. Given a basis $\left(u_{0},\ldots ,u_{n}\right)$ of functions defined on *I* and a set of data points, $p_{1},\ldots ,p_{l+1}$, corresponding to parameters $t_{1},\ldots ,t_{l+1}$, the objective of interpolation is to find a curve,

(1) $$\gamma \left(t\right):=\sum_{i=0}^{n}P_{i+1}u_{i}\left(t\right),\;t\in I,$$

which passes through the given data points; that is, $\gamma \left(t_{i}\right)=p_{i}$, i=1,…,l+1 (see Figure 1). The points $P_{1},\ldots ,P_{n+1}$ are called control points. The polygon $P_{1}\cdots P_{n+1}$, whose vertices are the control points, is called the control polygon.

It is worth noting that the number of control points $P_{1},\ldots ,P_{n+1}$ necessary to define the curve is equal to the number of interpolated data points (l=n). A better approximation to the curve to be fitted would be expected by increasing the number of given data points. Nevertheless, increasing the number of data points increases the number of control points and, thus, the complexity of the curve and computation.

**Figure 2:**
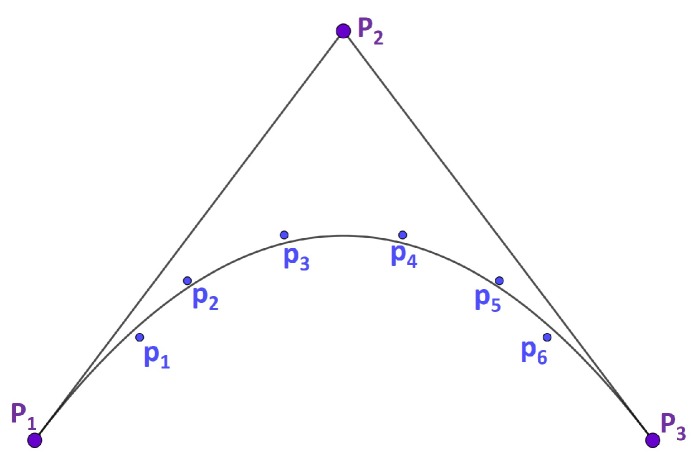
Curve fitting by least squares approximation, where $p_{1},p_{2},p_{3},p_{4},p_{5},p_{6}$ are the given data and $P_{1},P_{2},P_{3}$ are the control points.

On the other hand, an approximation curve is a curve that approximately fits the given data points but does not necessarily include them (see Figure 2). The most common technique for finding such curves is known as least squares approximation. It should be noted that, in an approximation, the number of control points that define the approximation curve are fewer than the number of given data points (l>n). Therefore, when fitting the same set of given data points $p_{1},\ldots ,p_{l+1}$, an approximation curve requires less computation than an interpolating curve representation (see Figure 2).

## 3. Materials and Methods

Now that the basis of curve definition has been explained, we first present an overview of the NanoPla. The NanoPla is a nanopositioning system, which is used to perform the experimental analysis of trajectory definition errors. Then, we define the analysis procedure which is utilized in this work for the comparison of the novel curve fitting method with traditional methods.

### 3.1. Nanopositioning Platform (NanoPla)

**Figure 3:**
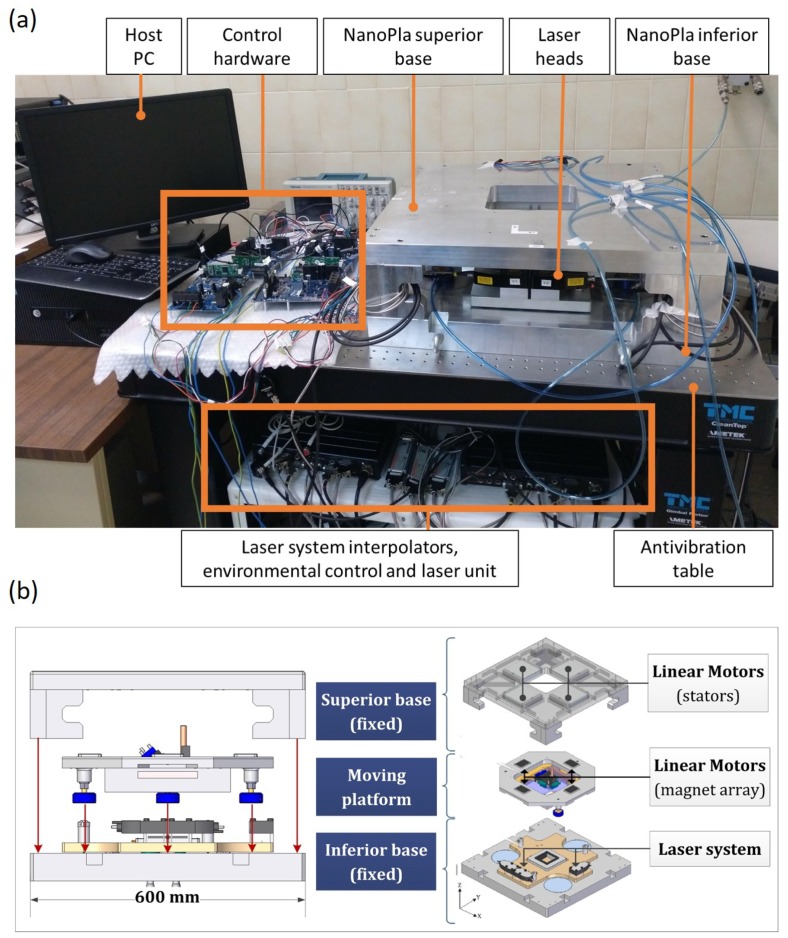
(**a**) Photograph of the NanoPla and the control system components; (**b**) front and exploded view of the NanoPla.

The NanoPla consists of three stages: a superior and an inferior base, both of which are fixed, and a moving platform placed in the middle (Figure 3). Three air-bearings levitate the moving platform, while Halbach linear motors generate the required forces for planar motion. A 2D laser system works as a positioning sensor, which provides positioning feedback in the X- and Y-axes and rotation around the Z-axis [3]. The control strategy presented in [14] was adapted and implemented in the NanoPla, in order to minimize the errors introduced by the control hardware and software. The resultant positioning control system of the NanoPla performs the motion and allows accurate positioning over a large range, up to 50 mm × 50 mm.

The NanoPla has been designed to work together with different kinds of tools and probes in various applications, such as metrology or nanomanufacturing. Some of these applications require the NanoPla to follow a specific trajectory; for example, one previously designed in a CAD/CAM system. Nevertheless, defining complex curve trajectories in traditional CAD/CAM systems results in trajectory definition errors, which can highly increase the final positioning error of the NanoPla. In Section 5, the positioning uncertainty of the NanoPla positioning system is calculated, in order to confine the trajectory definition errors such that they do not significantly affect the final positioning error.

### 3.2. Analysis Procedure

In this work, we analyze the errors in the definition of a trajectory determined by fitting a set of given data points $p_{1},\ldots ,p_{l+1}$. These given data points belong to a curve which was chosen to be a parametric curve, such that the resultant trajectory can be compared with the nominal values of the curve. The selected parametric curve is a cycloid, where the components x(t), y(t) take the form:

(2) $$\left\{\begin{array}{cc} x(t):=r(t-\sin t), & t\in [0,2\pi ]\\ y(t):=r(1-\cos t). \end{array}\right.$$

Cycloids are commonly used geometric figures in manufacturing applications (e.g., as gear tooth geometry). The cycloid is a transcendental curve and, thus, cannot be expressed by polynomials exactly. For this reason, cycloids cannot be incorporated into most commercial CAD systems [15].

**Figure 4:**
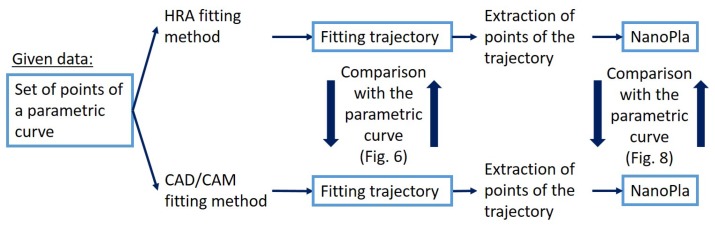
Diagram of the procedure for the analysis of the methods.

The rest of the paper is organized as follows: first, the HRA method is presented in Section 4. Then, the positioning uncertainty of the NanoPla control system is assessed in Section 5. Knowing the positioning uncertainty of the system allows us to confine the error in the trajectory definition (i.e., fitting error), such that it would not have a significant influence on the total NanoPla positioning error. Then, in Section 6, curve fitting is applied to a determined set of data points, $p_{1},\cdots ,p_{l+1}$, belonging to a cycloid of radius 1 mm, obtained for equidistant parameters on the interval t∈(0,2π). Curve fitting is performed by interpolation and least-squares approximation using two different methods: the HRA method, as described in Section 4, and in commonly used commercial CAD/CAM software. The resultant curve obtained by linear interpolation, the most commonly used interpolation method in numerical control (NC), is also included in the comparison. In addition, the resultant fitting errors are related to the number of control points required for the curve construction in each case, due to their relation to the final computational complexity. The target of Section 6 is to confirm that the HRA method is capable of satisfying the precision requirements with a minimum quantity of data points in the interpolation case, and with a minimum number of control points in the case of the least-squares approximation, in contrast to CAD/CAM methods. Finally, in Section 7, the relevance of the curve fitting errors in the total positioning error of the NanoPla is experimentally studied. For the experiments, the NanoPla is set to stop at certain positions on the trajectories defined by interpolating a set of points, either with the HRA method or by using CAM software. The contribution of the fitting errors to the final position of the NanoPla in each case is assessed. Figure 4 presents a diagram of the procedure followed in this work.

## 4. Accurate Curve Fitting with Shape-Preserving Representations: HRA Method

In this section, the HRA method for curve fitting is presented. Section 4.1 introduces HRA and a factorization of square strictly totally positive (STP) matrices, which allows for solving HRA linear systems with these matrices. Section 4.2 introduces the class of fg-Bernstein bases and the mentioned bidiagonal factorization of their STP collocation matrices. Section 4.3 presents a procedure for accurate curve fitting by means of interpolation and least-squares approximation using algorithms for solving linear systems with HRA.

### 4.1. High Relative Accuracy and Bidiagonal Factorizations

Let us recall that a quantity *X* can be obtained with HRA if the relative error of the computed value $\tilde{X}$ can be bounded, as follows:

$$\frac{||X-\tilde{X}\left|\right|}{\left||X|\right|}<Cu,$$

where *C* is a positive constant independent of the arithmetic precision and *u* is the unit round-off. An algorithm can be computed with HRA when it only uses products, quotients, the addition of numbers with the same sign, or subtraction of initial data (see [16]). Performing an algorithm with HRA is a very desirable goal. HRA implies that the relative errors of the computations are of the order of the machine precision, independent of the size of the problem. This goal is difficult to assure, although, in recent years, there have been some advances; in particular, in the field of numerical linear algebra. Let us recall that a matrix is totally positive (TP) if all of its minors are nonnegative and it is STP if all of its minors are positive (see [17]). Up until now, computations with HRA have been guaranteed only for a few classes of STP matrices. Previously, a reparametrization of the matrices was needed.

Neville elimination is a particularly important procedure when studying TP matrices. Neville elimination is an alternative to Gaussian elimination and makes zeros in a column of a matrix by adding to a given row the previous one multiplied by an appropriate coefficient called a multiplier (see [18,19,20]). Neville elimination has been used to characterize TP and STP matrices (see [18,19]). From Theorem 4.1 of [18] and p. 116 of [19], a given matrix *A* is STP if and only if the Neville elimination of *A* and $A^{T}$ can be performed without row exchanges, all the multipliers of the Neville elimination of *A* and $A^{T}$ are positive, and all the diagonal pivots of the Neville elimination of *A* are positive.

According to [19], together with the arguments of p. 116 of [19], an STP matrix $A\in R^{\left(n+1)\times (n+1\right)}$ can be factorized into the form

(3) $$A=F_{n}F_{n-1}\cdots F_{1}DG_{1}\cdots G_{n-1}G_{n},$$

where $F_{i}$ and $G_{i}$ are the lower and upper triangular bidiagonal matrices of the form

(4) $$\begin{array}{c} F_{i}=\left(\begin{array}{ccccccc} 1 & & & & & & \\ 0 & 1 & & & & & \\ & \ddots & \ddots & & & & \\ & & 0 & 1 & & & \\ & & & m_{i+1,1} & 1 & & \\ & & & & \ddots & \ddots & \\ & & & & & m_{n+1,n+1-i} & 1 \end{array}\right),G_{i}^{T}=\left(\begin{array}{ccccccc} 1 & & & & & & \\ 0 & 1 & & & & & \\ & \ddots & \ddots & & & & \\ & & 0 & 1 & & & \\ & & & {\hat{m}}_{i+1,1} & 1 & & \\ & & & & \ddots & \ddots & \\ & & & & & {\hat{m}}_{n+1,n+1-i} & 1 \end{array}\right) \end{array}$$

and $D=\mathrm{diag}\left(p_{1,1},\ldots ,p_{n+1,n+1}\right)$. The entries $m_{i,j}$ and ${\hat{m}}_{i,j}$ are the multipliers of the Neville elimination of *A* and $A^{T}$, respectively, and the diagonal entries $p_{i,i}$ are the diagonal pivots of the Neville elimination of *A*. In fact, a unique bidiagonal factorization can be obtained for nonsingular TP matrices (see [19,20]).

Bidiagonal factorizations have played a crucial role in deriving algorithms with HRA for STP matrices. When the bidiagonal factorization of the considered matrix is obtained with HRA, the computation of the inverse matrix, its eigenvalues and singular values, the solutions of some linear systems, and the computation of its QR factorization can be also performed with HRA, using the algorithms presented by Koev in [16,21].

Table 1 illustrates the accuracy of the computed solutions of Ax=c when using Koev’s algorithm with the bidiagonal factorization of *A*. For different values of *n*, collocation matrices $A_{n}$ of Bernstein polynomials of degree *n* on [0,1] corresponding to

(5) An:=n−1j−1tij−1(1−ti)n−j1≤i,j≤n,0<t1<⋯<tn<1,

have been considered at equidistant parameters. The solution of the systems $A_{n}x=c_{n}$, where $c_{n}$ is a vector formed by random numbers, have been obtained. The linear systems have also been solved by using Mathematica with a precision of 100 digits; this solution was considered to be exact. Furthermore, two approximations have been computed with Matlab, the first one using the Matlab command \ and the second one using Koev’s algorithm with the bidiagonal factorization of $A_{n}$.

As shown in Table 1, the accuracy of the obtained results when using the bidiagonal factorization of the coefficient matrix and Koev’s algorithm does not depend on the dimension of the considered problem. It is much better than the precision obtained when using the usual resolution with the Matlab command \.

### 4.2. The Class of Fg-Bernstein Bases

In CAGD, polynomial curves are usually represented in terms of the Bernstein basis of the space of polynomials of degree less than or equal to *n*, defined on I=[a,b] by

(6) (B0n,…,Bnn),Bkn(t):=nkt−ab−akb−tb−an−k,t∈I,k=0,…,n.

In [13], this basis was generalized as follows: let us suppose that f,g:I→R are nonnegative continuous functions. For a given n∈N, the corresponding fg-Bernstein basis of order *n* is defined as

(7) (u0n,…,unn),ukn(t):=nkfk(t)gn−k(t),t∈I,k=0,…,n.

Theorem 2 of [13] proves that, given nonnegative functions f,g:I→R such that f(t)≠0, g(t)≠0, ∀t∈(a,b), and f/g is a strictly increasing function, then

(8) A:=nj−1fj−1(ti)gn−j+1(ti)1≤i,j≤n+1,a<t1<⋯<tn+1<b

is STP. Moreover, in Theorem 3 of [13], the following bidiagonal decomposition (3) of the collocation matrices (8) was deduced

(9) $$A=F_{n}F_{n-1}\cdots F_{1}DG_{1}\cdots G_{n-1}G_{n},$$

where $F_{i}$ and $G_{i}$, 1≤i≤n, are lower and upper triangular bidiagonal matrices of the form (4) and $D=\mathrm{diag}\left(p_{1,1},\ldots ,p_{n+1,n+1}\right)$. The entries $m_{i,j},{\hat{m}}_{i,j}$, and $p_{i,i}$ are given by

(10) mi,j=gn−j+1(ti)g(ti−j)gn−j+2(ti−1)∏k=1j−1f(ti)g(ti−k)−f(ti−k)g(ti)∏k=2jf(ti−1)g(ti−k)−f(ti−k)g(ti−1),m^i,j=n−i+2i−1f(tj)g(tj),1≤j<i≤n+1,pi,i=ni−1gn−i+1(ti)∏k=1i−1g(tk)∏k=1i−1f(ti)g(tk)−f(tk)g(ti),1≤i≤n+1.

Let us observe that a sufficient condition to obtain the bidiagonal decomposition of *A* with HRA is that the expressions $f(t_{i})$, $g(t_{i})$, and $f\left(t_{i}\right)g\left(t_{k}\right)-f\left(t_{k}\right)g\left(t_{i}\right)$, for all k<i, can be computed with HRA.

There are many interesting choices of functions *f* and *g* allowing the definition of fg-Bernstein bases whose collocation matrices can be factorized as in (9) (see, e.g., Section 3 of [13]).

### 4.3. Curve Fitting with Fg-Bernstein Bases

This section presents an accurate method to solve interpolation and approximation problems using the fg-Bernstein bases introduced in Section 4.2.

The approach of the problem is as follows. Given I=[a,b] and a set of parameters $a<t_{1}<\cdots <t_{l+1}<b$, it is desirable to compute a function

(11) p(t):=∑j=1n+1cjnj−1fj−1(t)gn−j+1(t),t∈I,

expressed in terms of a fg-Bernstein basis of order *n*, as defined in (7), such that

$$p\left(t_{i}\right)=p_{i},\;1\leq i\leq l+1.$$

In order to compute the coefficients of p(t) with respect to the considered fg-Bernstein basis, the linear system Ac=p has to be solved, where

(12) A:=nj−1fj−1(ti)gn−j+1(ti)1≤i≤l+1;1≤j≤n+1

is the collocation matrix of the fg-Bernstein basis corresponding to the nodes $t_{1}<\cdots <t_{l+1}$, $p={\left(p_{1},\ldots ,p_{l+1}\right)}^{T}$ is the data vector, and $c={\left(c_{1},\ldots ,c_{n+1}\right)}^{T}$ is the vector with the coefficients to be calculated. Using Theorem 2 of [13], we can easily deduce that *A* is STP.

Let us observe that, in an interpolation problem, l=n; that is, the number of data points $\left(p_{1},\ldots ,p_{l+1}\right)$ is equal to the number of the coefficients $\left(c_{1},\ldots ,c_{n+1}\right)$. Moreover, the coefficient matrix is nonsingular and the linear system has a unique solution.

Now, let us describe how to solve this interpolation problem by means of the bidiagonal decomposition (8) of the collocation matrix of the fg-Bernstein basis.

In [16], an efficient algorithm for computing the solution of systems Ac=p was presented. In [21], the library “TNSolve” was made available, which contains an implementation of the mentioned algorithm for use with Matlab (or Octave). Its computational cost is $O(n^{2})$ elementary operations and requires, as input arguments, the bidiagonal factorization (3) of the matrix *A* and the vector *p* of the linear system Ac=p. Furthermore, a Matlab (or Octave) function, named “TNBDA”, has been implemented, which takes as input arguments a sequence of parameters $a<t_{1}<t_{2}<\cdots <t_{l+1}<b$ and computes the bidiagonal factorization (9) of the collocation matrix *A*. Section 6.1 shows the accuracy of the computed solutions of Ac=p when using the function “TNSolve” with the bidiagonal factorization of the collocation matrix of the fg-Bernstein basis given by “TNBDA”.

On the other hand, let us observe that, in an approximation problem, l>n; that is, the number of data points $\left(p_{1},\ldots ,p_{l+1}\right)$ is greater than the number of the coefficients $\left(c_{1},\ldots ,c_{n+1}\right)$. In this case, the polynomial, p(t), given in (11), is the solution to the least squares problem if it minimizes the sum of the squares of the deviations from the data

$$\sum_{i=1}^{l+1}{\left|p_{i}-p\left(t_{i}\right)\right|}^{2}.$$

Computing the coefficients $c_{j}$ of this polynomial p(t) is equivalent to solving, in the least squares sense, the overdetermined linear system Ac=p, where *A* is defined in (12) and $p={\left(p_{1},\ldots ,p_{l+1}\right)}^{T}$ is the data vector.

The unique solution of the problem is given by

(13) $$A^{T}Ac=A^{T}p.$$

Solving the previous normal Equation (13) is a worse-conditioned problem than computing the solution through the QR decomposition of the coefficient matrix *A*, which is the usual approach. Now, following the approach of [22], let us describe how to solve the approximation problem by means of the bidiagonal decomposition of the collocation matrix of the fg-Bernstein basis.

In [16], an efficient algorithm for computing the QR decomposition of an STP matrix *A* was presented. In [21], the Matlab (or Octave) library “TNQR”, containing an implementation of the above-mentioned algorithm, is available. Assuming that the bidiagonal factorization of *A* is known, “TNQR” computes the matrix *Q* and the bidiagonal factorization of the matrix *R* with HRA. Now, following the approach of [22], let us describe how to solve the least squares problem by means of a bidiagonal decomposition for rectangular matrices that generalizes the bidiagonal factorization described before when l=n (i.e., in the square case) and the QR decomposition provided by “TNQR”.

In order to compute the solution of the least squares problem, let us define the $R^{\left(l+1)\times (n+1\right)}$ matrix *M* such that

$$\begin{array}{cc} \; & M_{i,i}:=p_{i,i},\;\;i=1,\ldots ,n+1,\\ \; & M_{i,j}:=m_{i,j},\;j=1,\ldots ,n+1;\;\;i=j+1,\ldots ,l+1,\\ \; & M_{i,j}:={\hat{m}}_{i,j},\;i=1,\ldots ,n;\;\;j=i+1,\ldots ,n+1, \end{array}$$

where the $m_{i,j}$, ${\hat{m}}_{i,j}$, and $p_{i,i}$ are obtained as in (10). Then, using “TNQR”, the QR decomposition of *A* can be obtained, such that

$$A=Q\left(\begin{array}{c} R\\ 0 \end{array}\right),$$

where $Q\in R^{\left(l+1)\times (l+1\right)}$ is an orthogonal matrix and $R\in R^{\left(n+1)\times (n+1\right)}$ is an upper triangular matrix with positive diagonal entries. According to Section 1.3.1 in [23], the solution of the least squares problem is obtained from

(14) $$\left(\begin{array}{c} d_{1}\\ d_{2} \end{array}\right)=Q^{T}p,\;Rc=d_{1},$$

where $d_{1}\in R^{n+1}$, $d_{2}\in R^{l-n}$. The matrices *Q* and *R* have a special structure, as described in [24]. In particular, *R* is nonsingular and TP. In order to obtain the solution of the upper triangular system $Rc=d_{1}$, the routine “TNSolve” of [16] has been used, which performs the bidiagonal decomposition of the upper triangular TP matrix *R*. The numerical experiments in Section 6.2 show that the accuracy of the computed solutions does not depend on the size of the considered problem.

## 5. Positioning uncertainty of the NanoPla

The total NanoPla positioning error at a certain point of the trajectory ($e_{position}$) results from the sum of the positioning error of the system ($e_{NanoPla}$) and the error in the definition of the trajectory ($e_{trajectory}$), as represented in Equation (15)

(15) $$e_{position}=e_{NanoPla}+e_{trajectory}.$$

Therefore, it is convenient to analyze and calculate the positioning uncertainty of the NanoPla system, in order to confine the curve fitting error in the trajectory definition (Ce), such that it does not have a significant influence on the final positioning error.

**Figure 5:**
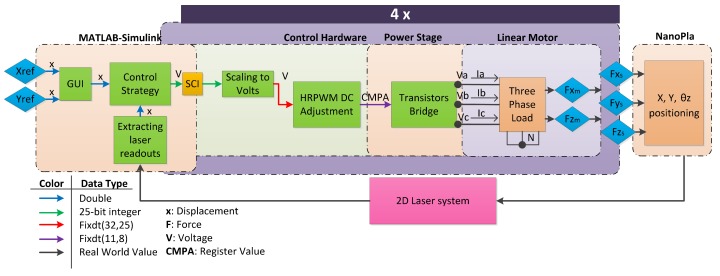
Scheme of the data flow in the NanoPla control system.

The NanoPla control system inputs are the X and Y coordinates of the desired position and the laser system readouts of the actual NanoPla position. In addition, rotation of the moving platform around the Z-axis is controlled to avoid laser system misalignment. The design and control system of the NanoPla has been optimized to correct systematic errors and minimize random positioning errors. The control strategy is computed in Simulink (Matlab), which outputs the phase voltages to be generated for each linear motor. These instructions are sent by a serial communication interface (SCI) to four digital motor control (DMC) kits of Texas Instruments. Each kit drives the phase voltages of one linear motor by pulse width modulation signals. The DMC kit works with 32-bit data and the input to the high-resolution PWM (HRPWM) module is a fixed point of 19 bits (11+8) bits. When the current flows through the motor’s coils, each motor generates a horizontal force and a vertical force. The four motors produce planar motion and are placed in parallel pairs; the first pair generates a force in the X-axis and the second pair in the Y-axis. These forces produce a displacement of the NanoPla, ultimately to the desired position. The vertical forces generated by the four motors help the air-bearings to levitate the platform. During computation of the control task, real numbers are represented with finite precision [25]. In Figure 5, a block diagram of the control system is represented, including the data type of the transmitted information, which affects the resolution of the system.

In Simulink, data are represented in double-precision floating-point format (64 bits) and, in this case, the rounding operation has no influence on the calculated results. The errors derived from the resolution of the data type at the SCI are also negligible. Nevertheless, the resolution of the HRPWM driving the phase currents contributes to the final positioning uncertainty. The other main contributors are the noise of the laser system, the noise of the generated currents, and the vibrations of the NanoPla. These errors cause root mean square (RMS) deviations of the positioning error in the open-loop, which can be experimentally measured. In addition, the geometrical errors of the 2D laser system have been characterized by a self-calibration procedure, as defined in [26], and corrected in the measurements. The uncertainty of the calibrated laser system also contributes to the final positioning uncertainty. Even though the laser system resolution is included inside the standard uncertainty of the laser system, its value is shown separately in the table, so it can be compared to the other contributor’s magnitudes. The calculation of the positioning uncertainty and its contributors are represented in Table 2, according to [27].

The resultant positioning uncertainty in the X–Y-plane, $U_{XY}$ (k=2), was equal to 0.50 μm over the working range of 50 mm × 50 mm. Additionally, it is worth mentioning that, when working in a closed-loop, the control system tends to correct the positioning error caused by the resolution of the system [28], which results in a difference between position feedback and position reference, generally smaller than 0.02 μm, and a RMS positioning noise, of approximately ±0.20 μm, whose exact values depend on the position and the controller configuration. The error introduced by the definition of the NanoPla trajectory should be confined, in order not to significantly increase the final positioning uncertainty. A confined fitting error of 0.05 μm or lower increases the final positioning uncertainty by less than 0.01 μm, which is considered acceptable for the requirements of the NanoPla.

## 6. Analysis of the Curve Fitting Errors

The target of this section is to perform curve fitting on a set of given data points using the HRA method and CAD/CAM software, in order to confirm that the HRA method provides a better fit. In this study, the curves that defined the trajectory of the NanoPla were calculated by curve fitting a set of data points $p_{1},\cdots ,p_{l+1}$ with equidistant parameter values $t_{1},\cdots ,t_{l+1}$, belonging to a cycloid curve of radius 1 mm, as defined in (2).

Curve fitting was performed using the HRA method and CAD/CAM systems with the requirement of obtaining a fitting error lower than 0.05 μm along the whole trajectory, in order to fulfill the NanoPla precision requirements without compromising the number of required data points and control points of the fitting curve. In the first subsection, the curves are obtained by interpolating sets of data points with different dimensions; that is, for different values of l+1. The smallest possible set of points required to fulfill the tolerance was obtained for each method. In the second subsection, the curves were obtained by performing least-squares approximations of sets of data points for different values of l+1 and the minimum number of control points required to fulfill the tolerance is obtained for each method. In both cases, the results obtained with the HRA method and with the commercial CAD/CAM software were analyzed.

### 6.1. Curve Fitting By Interpolation

As previously noted, interpolation consists of finding a curve through l+1 data points $p_{1},\ldots ,p_{l+1}$ with parameter values $t_{1},\ldots ,t_{l+1}$. For this reason, when interpolating through a set of points using an exact method, the interpolation errors at these points were zero. Nevertheless, at non-data intervals (data between interpolation points), the interpolation errors can be calculated as the difference between the nominal parametric curve and the interpolated curve, when the equation of the nominal curve is known. In addition, as mentioned in Section 2, in an interpolation problem, the number of control points equals the number of given data points (l=n). Thus, in this subsection, the number of data points and control points will be indistinctly referred to as n+1.

For comparison, the curve trajectory was generated by three exact interpolation methods: linear interpolation, CAD/CAM system interpolation, and the HRA method presented in Section 4. The interpolation results of some commonly used CAD and CAM systems have been compared and, even though their results slightly differed, the errors had similar magnitude; thus, the difference was not significant. Therefore, the results of CAM software have been used as a reference in this subsection. On the other hand, interpolation with the HRA method was performed using two different fg-Bernstein bases. One of them is the polynomial basis obtained with f(t)=t and g(t)=1−t, t∈[0,1] (HRA interpolation with this basis will be called HRA IB1) and the other one is the trigonometric basis obtained with $f\left(t\right)=\sin\left((\Delta +t)/2\right)$ and $g\left(t\right)=\sin\left((\Delta -t)/2\right)$ , t∈[−Δ,Δ] and Δ=1.57 (HRA interpolation with this basis will be called HRA IB2). Thus, interpolation with the HRA method was applied as follows: Firstly, for different values of n+1, collocation matrices at equidistant parameters in the interior of the interval domain of the selected fg-Bernstein bases were considered. Secondly, in order to obtain the n+1 control points, the linear system Ac=p was computed by applying the interpolation method explained in Section 4, when l=n. Knowing the control points, the parametric curve was evaluated and the deviations of the interpolated curve from the cycloidal nominal were obtained by computing the distance at equally distributed points along the profile curve.

**Figure 6:**
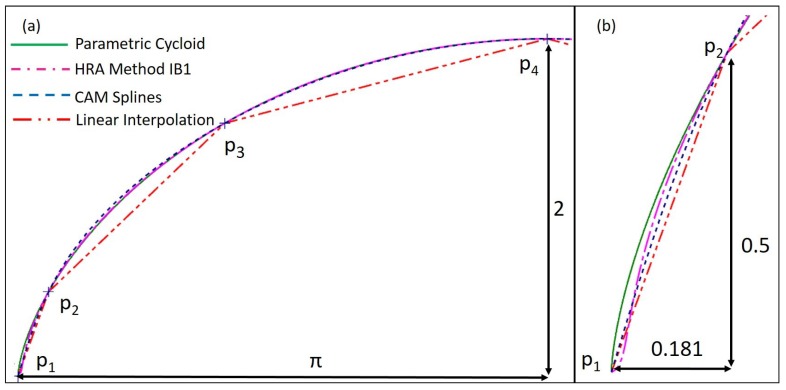
(**a**) Resultant interpolated curves obtained for n+1=7, represented for t∈(0,π); (**b**) resultant interpolated curves obtained for n+1=7, in the first non-data interval.

Figure 6a shows the resultant curves for the three interpolation methods, for n+1=7; that is, seven interpolation points and seven control points. For simplification and due to symmetry, only half of the curves are represented. As can be seen, the greatest interpolation errors occurred when applying linear interpolation. With this method, the maximum interpolation errors appear at the middle zone of the intervals, whose interpolating points were further from each other (interval between $p_{3}$ and $p_{4}$). Nevertheless, when applying CAM/CAD system spline interpolation and the HRA method, the interpolation errors were reduced. In both cases, the maximum interpolation errors appeared in the middle zones of the first intervals. The first non-data interval is shown in Figure 6b.

**Figure 7:**
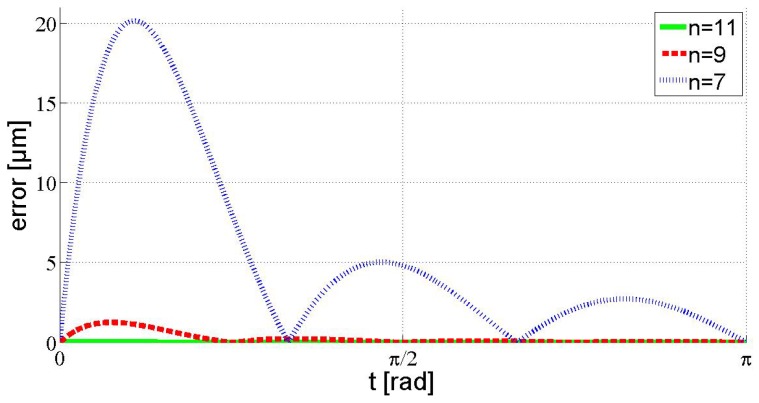
Interpolation errors obtained by the high relative accuracy (HRA) method IB1.

The CAD and CAM systems spline bases are usually unknown for the user. In other works, such as [29], the interpolation algorithm had to be experimentally extracted to simulate the interpolation errors. In this work, the interpolation errors were calculated graphically in the CAM program. However, the interpolation errors of linear interpolation and the HRA method were calculated analytically. In both cases, the errors of the interpolated curve from the cycloidal nominal were obtained by computing the distance at equally distributed points along the profile curve. The interpolation errors obtained by the HRA method IB1, along the whole range of the cycloid, are represented in Figure 7, for n+1=7, 9, and 11. As shown, the maximum interpolation errors occurred in the first interval, then decreased by more than half.

In Table 3, the fitting errors of the interpolated curves for the number of interpolated points n+1=7, 9, 11, and 21 are represented. It was observed that, for this specific curve, the first and second non-data intervals were the most critical, because they contained the greatest fitting errors when interpolating with CAM system splines, as well as with the HRA method. For this reason and for simplification, only these intervals are represented in Table 3. As can be seen, linear interpolation always presented the worst results. In addition, for the same number n+1 of interpolation points, the HRA method presented a considerably smaller error than the CAM system splines. When interpolating with HRA method IB1, for a number of interpolation points higher than 11, the resultant errors became negligible for the NanoPla application (Ce < 0.05 μm); while, in the HRA method IB2, 16 interpolation points were required. In contrast, it has been verified that more than 49 interpolation points are necessary to achieve an error lower than 0.05 μm at the non-data intervals, when interpolating with CAM/CAD system splines, and more than 250 interpolation points are needed when applying linear interpolation. Therefore, it can be inferred that the HRA method is capable of achieving accurate curve fitting while interpolating a smaller number of data points, in comparison with commonly used CAD/CAM systems.

### 6.2. Curve Fitting by Least Squares Approximation

Least-squares approximation is not an exact method; that is, the resultant fitting curve does not necessarily contain the given data points, but only approximately fits them. In an approximation problem, the number of control points can be externally defined and is directly related to the fitting errors. As mentioned above, the number of control points defines the complexity of the fitting curve: typically, a smaller number of control points results in a simpler curve, but also in greater fitting errors. Nevertheless, an elevated number of control points does not assure accurate fitting, as some control points may be redundant or inadequate [10]. In addition, in an approximation problem, the fitting tolerance is defined as the maximum permitted error between the given data points and the resultant approximation curve. Nevertheless, the error between the smooth curve and the nominal curve can be greater over non-data intervals. For this reason, the dimension l+1 of the set of given data points has to be large enough such that the fitting error does not increase significantly over the non-data intervals.

For comparison, curve trajectories were generated by two least-squares approximation methods: CAD/CAM system approximation and the HRA method presented in Section 4. In CAD/CAM systems, when curve fitting by approximation, a tolerance for the fitting error must be defined by the designer. The smaller this tolerance is, the greater the number of control points the approximation curve requires. Similar to the interpolation operation, the approximation operation was performed using some of the most commonly used CAM/CAD systems and the results were compared. Even though the obtained results were similar, some programs presented more information to the user about the approximation function than others. For example, in some programs the approximation spline degree can be selected, the points in the curve where the maximum fitting error occurs are shown, and the magnitude of the consequent error, along with the magnitude of the average fitting error along the whole curve, is calculated. In this section, the results of CAD software which calculated the maximum fitting error (confined error, Ce) and graphically showed its location was used as a reference. On the other hand, the least squares approximation with the HRA method was performed using two different fg-Bernstein bases, the same as those in the previous subsection. One of them is the polynomial basis obtained with f(t)=t and g(t)=1−t, t∈[0,1] (HRA least-squares approximation with this basis will be called HRA LSB1), and the other one is the trigonometric basis obtained with $f\left(t\right)=\sin\left((\Delta +t)/2\right)$ and $g\left(t\right)=\sin\left((\Delta -t)/2\right)$ , t∈[−Δ,Δ] and Δ=1.57 (HRA least-squares approximation with this second basis will be called HRA LSB2). The least squares approximation with the HRA method was carried out as follows: first, for different values of l+1, collocation matrices at equidistant parameters in the interior of the interval domain of the selected fg-Bernstein bases were considered. Second, in order to obtain the n+1 control points, the linear system Ac=p was computed by applying the interpolation method explained in Section 4, with l>n. Knowing the control points, the parametric curve was evaluated and the maximum fitting error of the approximated curve from the cycloidal nominal was obtained as the confined error, by computing the maximum distance at equally distributed points along the profile curve.

According to the positioning accuracy of the NanoPla, the fitting tolerance was set to be smaller than 0.05 μm for the approximation operation in every case. Table 4 represents the number of control points that were required to generate a fitting curve from the given data points, $p_{1},\ldots ,p_{l+1}$, by least squares approximation, with the reference CAD method and the HRA method. The resultant confined error (Ce)—that is, the maximum fitting error—obtained for every method is also represented in the table. In the case of the CAD method, the confined error was calculated and provided by the software. For the HRA method, the confined error was analytically calculated. In addition, the results of the HRA method are shown for the two different bases, HRA LSB1 and HRA LSB2.

It can be observed that, in every case, with the same set of given data points, the HRA methods were capable of obtaining an approximation curve within a tolerance of 0.05 μm with fewer control points, in comparison with the CAD method. In addition, it is worth noting that, for the HRA methods, when the number of given data points increased, the number of control points required to fulfill the tolerance constraint remained practically constant, in contrast to the CAD method. Thus, it can be inferred that the HRA method provided a better approximation, requiring fewer control points, and that it did not define redundant control points.

## 7. Experimental Results

The previous section showed that the HRA method is capable of performing accurate curve fitting requiring fewer data points in the interpolation operation and with a minimum number of control points in a least-squares approximation, in comparison with CAD/CAM systems. The resultant fitting curve can be used to define the trajectory of a positioning system, such as the NanoPla. This section experimentally analyzes the relevance of the trajectory definition errors in the final positioning error of the NanoPla when the trajectory is defined by fitting a certain set of data points.

The NanoPla was required to displace itself to positions along a defined trajectory with minimum positioning error. As was assessed in Section 5, the positioning uncertainty $U_{XY}$ (k=2) of the NanoPla was 0.50 μm. Therefore, trajectory definition errors lower than 0.05 μm were considered to be negligible in this application. In order to analyze the contribution of the trajectory definition errors to the final positioning error of the NanoPla, the trajectory was defined by interpolating a set of points using both the CAM/CAD system methods and the HRA method. Then, the position of the NanoPla at certain points of the trajectories was compared to the nominal curve trajectory position.

In Section 6, it was stated that the interpolating errors when calculating a cycloid (r = 1 mm) based on 11 points were lower than 0.05 μm, when using the HRA method. Nevertheless, the errors were up to 4.5 μm when interpolating with CAM system splines. In this experiment, the trajectory of the NanoPla was defined by the curves resulting from interpolating a set of 11 points of a parametric cycloid (r = 1 mm) separated by equal intervals of *t* (△t=2π/10 rad). The interpolation was performed by CAM system splines and by the HRA method, in order to verify the interpolating error effect when implementing the trajectories in the NanoPla. In order to observe the effect of the positioning noise of the NanoPla in a closed-loop, the control system of the NanoPla was set to keep the moving platform still at certain positions in the trajectories. The resultant X,Y positions of the NanoPla were experimentally recorded for 30 s, with a readout taken every 0.12 seconds.

**Figure 8:**
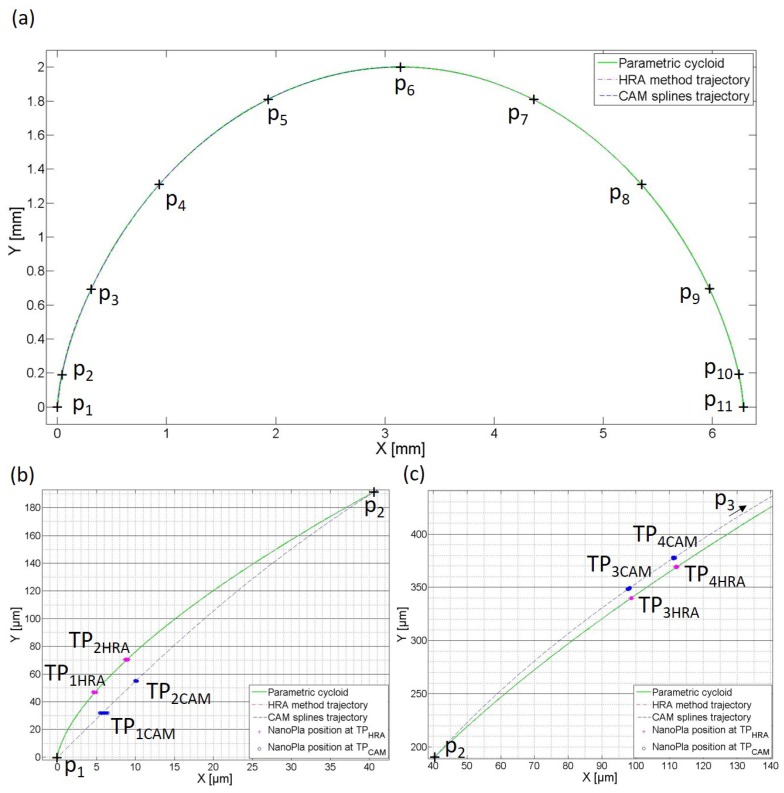
(**a**) Trajectories defined by interpolating the data points $p_{1},\ldots ,p_{11}$ with the HRA method and CAM splines; detail of the first non-data interval (**b**) and the second non-data interval (**c**), including the position of the NanoPla at certain trajectory points.

Figure 8 represents the curve trajectories defined by interpolating 11 data points ($p_{1},\ldots ,p_{11}$) with the HRA method and with CAM splines; in addition, the parametric curve is also included. Due to the fact that the fitting errors occurred at a micrometer scale, they cannot be observed in Figure 8a. For clarification, Figure 8b,c show an amplification of the first and second non-data intervals, where the greatest fitting errors take place. Figure 8b,c also include the 250 experimental readouts of the laser system which represents the positions of the platform where the control system was set to stay still at points belonging to the trajectory of the curve, as defined by the HRA method ($TP_{1HRA}$ , $TP_{2HRA}$, $TP_{3HRA}$, and $TP_{4HRA}$; magenta in the figure) and by CAM system splines ($TP_{1CAM}$, $TP_{2CAM}$ , $TP_{3CAM}$, and $TP_{4CAM}$; blue in the figure). These trajectory points (TP) were selected in the non-data intervals of the curve, where the greatest fitting errors occurred, as can be seen in the figures. Therefore, they present a worst-case scenario.

In Figure 8b,c, it can be seen that the HRA method trajectory was almost coincident with the parametric curve, while the CAM trajectory presented a larger fitting error. At the trajectory points (TP) where the NanoPla was set to keep still, the positioning error was the result of the sum between the positioning error of the NanoPla and the errors in the definition of the trajectory (Equation (15)), which were the curve fitting errors. In Table 5, the NanoPla positioning error and the trajectory definition error at each trajectory point, as well as the total positioning error, are represented. The NanoPla positioning error at the TPs is expressed as the sum of a constant value and a standard deviation. The constant value is the difference between the laser system position feedback and the reference position in the controller, calculated by averaging all laser readouts at the TP. The standard deviation includes the RMS positioning noise and the calibrated laser system uncertainty.

The interpolating errors for $TP_{1HRA}$ and $TP_{2HRA}$ were 0.04 μm and 0.03 μm, respectively; as can be seen from Figure 8b, they did not significantly affect the final positioning accuracy. On the contrary, the interpolating errors of $TP_{1CAM}$ and $TP_{2CAM}$ were 3.1 μm and 3.8 μm, respectively, and they had a significant effect in the total positioning error of the NanoPla, being the greatest contributor. Similarly, in the second non-data interval (Figure 8c), the interpolating error for $TP_{3HRA}$ and $TP_{4HRA}$ was 0.007 μm in both cases, which was negligible in comparison to the NanoPla positioning error. The interpolating errors of $TP_{3CAM}$ and $TP_{4CAM}$ were 4.5 μm in both cases. As in the first interval, they were the greatest contributor to the final positioning error of the NanoPla.

## 8. Conclusions

In nanopositioning systems, such as the NanoPla, the final positioning accuracy can be affected by errors in the definition of the trajectory. Hence, this work proposed the use of a novel method for the parametric representation of curves, allowing for curve fitting with high relative accuracy for the definition of curve trajectories. This method, which uses collocation matrices of fg-Bernstein bases, is applicable in CAGD and greatly improves the accuracy in trajectory definition by curve fitting, when compared to traditional CAD/CAM methods implemented in CAD/CAM software.

In this work, the positioning uncertainty of the NanoPla (k=2) was calculated to be 0.50 μm. Thus, the trajectory definition errors were required to be lower than 0.05 μm, in order not to significantly affect the final positioning accuracy of the system. Sets of given data points were curve fitted by interpolation and least-squares approximation, using both the proposed HRA method and CAD/CAM software, with the requirement of fulfilling the tolerance of 0.05 μm. Finally, it was experimentally verified that, when defining the trajectory by interpolating a given set of 11 data points with the HRA method, the fitting errors at non-data intervals were negligible, in comparison to the NanoPla positioning uncertainty. In contrast, when defining the trajectory with CAM splines with the same set of 11 interpolation points, the interpolation errors were the greatest contributors to the NanoPla final positioning error. This work focused on the contribution of the trajectory definition errors to the final positioning accuracy of the NanoPla. In future work, the dynamic response of the NanoPla when performing a trajectory of motion will be studied.

The importance in this work of the fg-Bernstein bases arises from the fact that their collocation matrices admit many algebraic computations with HRA. In particular, the proposed HRA method can be applied to perform interpolation and least-squares approximation with high precision and can be considered as a modeling tool for trajectories. The benefits of the proposed HRA method that have been shown in this paper are that it is capable of performing accurate interpolation with a minimum quantity of data points and, when performing a least-squares approximation, it is capable of obtaining an accurate approximation curve with a minimum number of control points. In addition, the number of obtained control points was independent of the size of the problem. On the contrary, in CAD/CAM systems, a higher number of data points was required for accurate curve fitting and the number of control points increased with the size of the problem, which may result in redundant or inadequate control points. As a result, the proposed HRA method offers a better positioning accuracy than commonly used CAD/CAM methods when defining a trajectory by curve fitting (either by interpolation or least-squares approximation). Hence, implementing the HRA method in CAD/CAM systems would be of great interest to nanomanufacturing applications in which a nanopositioning stage is required to follow a predefined trajectory.

## Figures and Tables

**Table 1:** Relative errors in solving $A_{n}x=c_{n}$ using the bidiagonal factorization of collocation matrices of Bernstein polynomials (5) and Koev’s algorithm.

n	$A_{n}\setminus c_{n}$	HRA
10	$3.3579\times 10^{-13}$	$1.1191\times 10^{-15}$
20	$1.2413\times 10^{-9}$	$6.2974\times 10^{-16}$
25	$4.1424\times 10^{-7}$	$2.0843\times 10^{-15}$
50	1.0000	$7.5480\times 10^{-15}$

**Table 2:** NanoPla positioning uncertainty contributors and calculation.

Source	Justification	Value
Resolution at the HRPWM $u_{HRPWM}$	Resolution of $2.62\times 10^{-5}$ V [14]	$700/\sqrt{12}$ nm
Laser system resolution $u_{Lres}$	Resolution of 1.58 nm	($1.58/\sqrt{12}$ nm)
Calibrated Laser system $u_{Lcal}$	Geometrical errors + measuring system calibration [26]	99 nm
RMS positioning error $u_{RMS}$	Laser system noise + phase currents noise + vibrations	110 nm
Positioning uncertainty $U_{XY}$ (k=2)	$U_{XY}\left(k=2\right)=k\sqrt{u_{HRPWM}^{2}+u_{Lres}^{2}+u_{Lcal}^{2}+u_{RMS}^{2}}$	501 nm

**Table 3:** Maximum interpolating errors for a curve generated based on a set of points $p_{1},\ldots ,p_{n+1}$ in the first and second non-data intervals, for different interpolation methods.

n+1	Intervals	Linear	CAM	HRA IB1	HRA IB2
7	(0,2π/6)	27.6μm	18.0 μm	20.17 μm	17.84 μm
7	(2π/6,4π/6)	93.5 μm	17.1 μm	5.31 μm	5.60 μm
9	(0,2π/8)	11.8 μm	8.0 μm	1.25 μm	3.95 μm
9	(2π/8,4π/8)	41.9 μm	8.5 μm	0.22 μm	0.93 μm
11	(0,2π/10)	6.1 μm	4.0 μm	0.05 μm	0.94 μm
11	(2π/10,4π/10))	22.0 μm	4.5 μm	<0.01 μm	0.18 μm
21	(0,2π/20)	0.7 μm	0.5 μm	≪1 nm	<0.01 μm
21	(2π/20,4π/20)	2.8 μm	0.6 μm	≪1 nm	<1 nm

**Table 4:** Number of control points ($P_{i}$, i=1,…,n+1) and maximum fitting errors (Ce) for a curve generated based on a set of points $p_{i}$, i=1,…,l+1, using different approximation methods.

l+1	CAD		HRA LSB1		HRA LSB2	
	n+1	**Ce**	n+1	**Ce**	n+1	**Ce**
51	21	0.045μm	10	0.004μm	12	0.046μm
101	38	0.006μm	11	0.008μm	13	0.037μm
251	44	0.023μm	11	0.013μm	14	0.017μm
501	34	0.046μm	11	0.015μm	14	0.021μm
1001	38	0.036μm	11	0.016μm	14	0.023μm

**Table 5:** NanoPla positioning system errors ($e_{NanoPla}$), trajectory errors ($e_{trajectory}$) and total positioning errors ($e_{position}$) at certain points of the trajectory (totally positive (TP)) defined by the HRA method and CAM system.

Trajectory Points	$e_{\mathrm{NanoPla}}$	$e_{\mathrm{trajectory}}$	$e_{\mathrm{position}}$
$TP_{1HRA}$	0.01±0.16μm	0.04μm	0.05±0.16μm
$TP_{1CAM}$	0.02±0.21μm	3.1μm	3.12±0.21μm
$TP_{2HRA}$	0.01±0.19μm	0.03μm	0.04±0.19μm
$TP_{2CAM}$	0.00±0.13μm	3.8μm	3.80±0.13μm
$TP_{3HRA}$	0.02±0.22μm	0.007μm	0.027±0.22μm
$TP_{3CAM}$	0.01±0.32μm	4.5μm	4.51±0.32μm
$TP_{4HRA}$	0.02±0.31μm	0.007μm	0.027±0.31μm
$TP_{4CAM}$	0.01±0.16μm	4.5μm	4.51±0.16μm
